# Coordinated protein co‐expression in plants by harnessing the synergy between an intein and a viral 2A peptide

**DOI:** 10.1111/pbi.12670

**Published:** 2017-03-30

**Authors:** Bei Zhang, Madhusudhan Rapolu, Sandeep Kumar, Manju Gupta, Zhibin Liang, Zhenlin Han, Philip Williams, Wei Wen Su

**Affiliations:** ^1^ Department of Molecular Biosciences and BioengineeringUniversity of Hawaii at ManoaHonoluluHIUSA; ^2^ Dow AgroSciences LLCIndianapolisINUSA; ^3^ Department of ChemistryUniversity of Hawaii at ManoaHonoluluHIUSA

**Keywords:** FMDV 2A, gene and trait stacking, intein, molecular farming, protein expression, production of protein complex

## Abstract

A novel approach is developed for coordinated expression of multiple proteins from a single transgene in plants. An *Ssp* DnaE mini‐intein variant engineered for hyper‐N‐terminal autocleavage is covalently linked to the foot‐and‐mouth disease virus 2A (F2A) peptide with unique ribosome skipping property, via a peptide linker, to create an ‘IntF2A’ self‐excising fusion protein domain. This IntF2A domain acts, *in cis*, to direct highly effective release of its flanking proteins of interest (POIs) from a ‘polyprotein’ precursor in plants. This is successfully demonstrated in stably transformed cultured tobacco cells as well as in different organs of transgenic tobacco plants. Highly efficient polyprotein processing mediated by the IntF2A domain was also demonstrated in lettuce and *Nicotiana benthamiana* based on transient expression. Protein constituents released from the polyprotein precursor displayed proper function and accumulated at similar levels inside the cells. Importantly, no C‐terminal F2A extension remains on the released POIs. We demonstrated co‐expression of as many as three proteins in plants without compromising expression levels when compared with those using single‐protein vectors. Accurate differential cellular targeting of released POIs is also achieved. In addition, we succeeded in expressing a fully assembled and functional chimeric anti‐His Tag antibody in *N. benthamiana* leaves. The IntF2A‐based polyprotein transgene system overcomes key impediments of existing strategies for multiprotein co‐expression in plants, which is particularly important for gene/trait stacking.

## Introduction

Coordinated expression of multiple proteins in plants is essential for unravelling fundamental cellular mechanisms as well as development of next‐generation crops with improved traits. By co‐expressing multiple proteins, crops have been successfully modified to acquire enhanced abiotic tolerance, improved pathogenic resistance, as well as enriched nutritional contents (Arvinth *et al*., 2010; Chen *et al*., 1998; Sun *et al*., 2012). Currently, only a limited number of approaches are available for co‐expression of multiple proteins in plants. Cotransformation of multiple monocistronic expression cassettes and crossing of different transgenic events containing single expression cassettes are the most prevalent approaches to create genetically modified crops with multiple stacked transgenes (James, 2010). However, these approaches require laborious screening, breeding and introgression processes. Moreover, coordinated expression of the resulting proteins often necessitates extensive tuning of the promoter and regulatory elements. Other notable techniques for multigene co‐expression or gene stacking include those that are based on polycistronic or polyprotein vectors. Gene stacking in plants using polycistronic transgenes mainly operates on internal translational initiation mediated by internal ribosome entry sites (IRES) (François *et al*., 2002b; Ha *et al*., 2010). However, IRES‐mediated translational initiation is less efficient compared with that of the 5′‐cap‐mediated initiation and results in uneven protein co‐expression (François *et al*., 2002b; Ha *et al*., 2010; Mizuguchi *et al*., 2000).

In co‐expression of multiple proteins from a polyprotein transgene, the constituent POIs are released from the polyprotein precursor during or after protein translation. Some of the polyprotein expression systems have exploited endogenous plant protease activity to liberate multiple POIs connected by protease substrate sequences (François *et al*., 2002a; Urwin *et al*., 1998; Walker and Vierstra, 2007; Zhang *et al*., 2011). However, processing of the polyproteins in this case can only occur in a particular cellular compartment within hosts where the specific proteases are located. Alternatively, incorporating an exogenous protease within the polyprotein system is used to overcome this problem. Some notable examples include polyprotein vectors that employ NIa protease recognition sequence together with the Nla proteinase from the tobacco etch virus (Marcos and Beachy, 1994, 1997) and a similar vector that employs a NIa proteinase from the tobacco vein mottling virus (Dasgupta *et al*., 1998). While proper polyprotein processing was achieved using these vectors, the protein expression levels were generally low. With the protease‐based polyprotein approaches, formation of a properly folded polyprotein precedes the proteolytic processing to release the individual protein units. This could be especially problematic as the number of proteins to be expressed increases which results in very large polyproteins. To this end, a strategy that involves cotranslational protein cleavage would be more desirable, to avoid potential misfolding of the large polyproteins.

Polyprotein expression based on the unique ribosome skipping mechanism of the FMDV 2A (F2A) peptide (Donnelly *et al*., 2001b) operates cotranslationally. The F2A peptide has been used to direct multiprotein co‐expression in a wide range of eukaryotic hosts (de Felipe, 2004; de Felipe *et al*., 2006). However, the ‘remnant’ 2A residues appended to the carboxyl terminus of the processed proteins could hinder protein activity and/or cellular targeting (François *et al*., 2004; Randall *et al*., 2004; Samalova *et al*., 2006). Removal of the extraneous 2A residues using host endogenous proteases has been attempted in plant (François *et al*., 2002a) and mammalian systems (Fang *et al*., 2005), yet the requirement of specific endogenous proteases and inability to completely avoid appending remnant protease substrate linker residues to the cleaved POIs have significantly limited its general usefulness. To resolve these problems, we have exploited *in vivo* self‐excision of the 2A sequence extension *via* intein‐mediated N‐terminal autocleavage, by fusing an engineered mini‐intein with the 2A sequence through a linker to create the ‘IntF2A’ self‐excising domain.

Inteins mediate protein splicing in which a portion of the protein excises itself while ligating flanking protein sequences. The protein splicing element is the ‘intein’, while the protein sequences flanking the intein sequence are termed ‘exteins’. By mutating the essential C‐terminal asparagine to alanine (N159A), inteins can be modified to boost their autocatalytic N‐terminal cleavage efficiency (i.e. cleave off protein flanking the intein's N‐terminus), with essentially diminished splicing activity (Amitai *et al*., 2009; Xu and Perler, 1996). The N‐terminal autocleavage efficiency can also be modulated by amino acid residues in the flanking extein regions.

Unlike other existing polyprotein vector technologies, the IntF2A‐based approach enables cotranslational ‘cleavage’ via 2A's translational recoding activity, followed by very efficient and rapid post‐translational autocatalytic cleavage *via* intein at its N‐terminal junction, and it does not require the presence of any host‐specific proteinases or cofactors. As such, this approach can potentially be applicable across a broad range of hosts. Also, the IntF2A‐mediated *in vivo* polyprotein autoprocessing is not affected by the subcellular location of the protein. The present work provides detailed characterization of the IntF2A‐based polyprotein expression system in plants for coordinating co‐expression of multiple functional proteins, differential cellular targeting of processed proteins and production of complex protein products (by demonstrating synthesis of a functional IgG antibody).

## Results

### Processing of the IntF2A‐based polyprotein in plants

**Figure 1:**
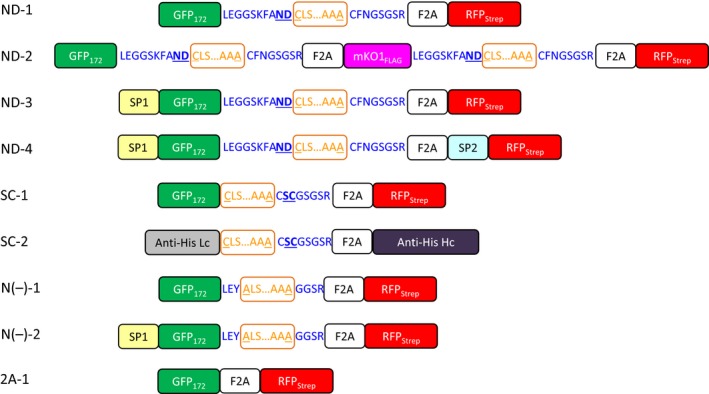
IntF2A‐based polyprotein cassettes investigated in this study. The intein domains are boxed and marked in orange. Intein terminal residues are underlined. Linker sequences are shown in blue wherein N‐terminal autocleavage accelerating flanking extein residues are shown in underline bold letters. F2A: 2A peptide from foot‐and‐mouth disease virus; SP1: *Arabidopsis thaliana* basic chitinase signal peptide; SP2: rice α‐amylase signal peptide. GFP_172_: Green fluorescent protein with an internal hexa‐histidine Tag between amino acid residues 172 and 173. RFP_S__trep_: monomeric cherry fluorescent protein with a C‐terminal Strep Tag. mKO1_FLAG_: monomeric Kusabira‐Orange 1 fluorescent protein with a C‐terminal FLAG Tag. Anti‐His Lc: light chain of anti‐His Tag antibody; Anti‐His Hc: heavy chain of anti‐His Tag antibody.

**Figure 2:**
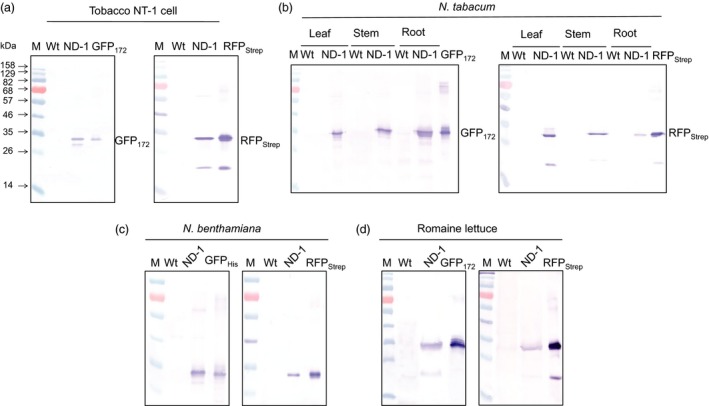
Characteristics of co‐expressing two fluorescent proteins from the IntF2A‐based polyprotein cassette ND‐1 in plants. Efficient *in vivo* autocleavage and release of the fluorescence reporters in (a) tobacco NT1 cells, (b) different organs of *Nicotina tabacum* plants, (c) leaf tissue of *Nicotina benthamiana* and (d) leaf tissue of Romaine lettuce, shown using Western blots probed with anti‐GFP (left panel) and anti‐Strep Tag (right panel) antibodies for detecting released upstream and downstream proteins of interest, respectively. Hereinafter, ‘M’ & ‘Wt’ denote molecular marker and nontransformed wild‐type control, respectively.

IntF2A‐based polyprotein cassettes (summarized in Figure 1) were assembled by connecting an upstream POI (POI1) and a downstream POI (POI2) with the intervening IntF2A autoprocessing domain that enables self‐excision at both terminal junctions (Figure S1). To maximize the 2A activity, a 58aa F2A sequence that includes 39 aa from the C‐terminal portion of the 1D capsid protein preceding the 2A was used (Donnelly *et al*., 2001a). After cotranslational F2A‐mediated release of POI2, POI1 can be liberated by the N‐terminal cleavage activity of the *Ssp* DnaE mini‐intein with an N159A mutation. Processing of the IntF2A‐based polyprotein in plants was initially characterized using Western blot analysis of the total protein extract from tobacco NT1 cells expressing the ND‐1 polyprotein cassette (Figure 1). As shown in Figure 2a, essentially complete release of both POIs, that is GFP_172_ and RFP_Strep_, was observed when the samples were probed with anti‐GFP or Strep Tag antibodies. The processed proteins migrated to the same position as purified protein standards (~28 kDa). The lower immunoreactive band on the Strep Tag Western blot resulted from hydrolysis of the acylimine bond at the RFP chromophore under the denaturing condition imposed by the sample heating step (Campbell *et al*., 2002).

**Figure 3:**
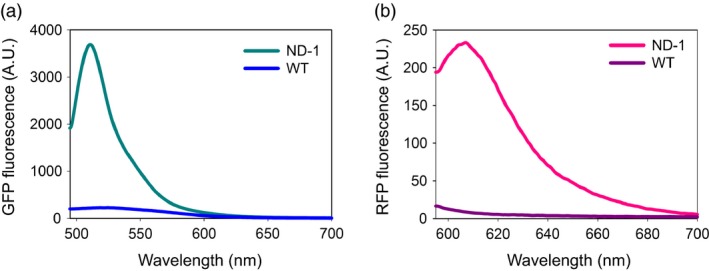
Processed proteins from ND‐1 in tobacco NT1 cells retain proper fluorescence property based on fluorescence spectroscopy measurement (a,b). Characteristic fluorescence spectra were detected in ND‐1 extracts but not in the untransformed wild‐type controls (WT).

Similar to undifferentiated tobacco NT1 cells, when the ND‐1 polyprotein was expressed in *Nicotina tabacum* cv. Xanthi plants, efficient release of both upstream GFP_172_ and downstream RFP_Strep_ was detected in leaf, stem and root extracts (Figure 2b). Aside from tobacco, efficient processing of the ND‐1 polyprotein was observed in *Nicotiana benthamiana* and Romaine lettuce (*Lactuca sativa* L. var. *longifolia*) based on transient expression *via* agroinfiltration (Figure 2c,d). These results support the general utility of the IntF2A polyprotein system in a wide range of plant species for efficient coordinated production of multiple proteins. When examined using fluorescence microscopy, tobacco NT1 cells expressing ND‐1 displayed bright fluorescence (Figure 7d). Characteristic GFP and RFP spectra, distinctive from the background autofluorescence of untransformed wild‐type control, were also observed in the protein extracts of transgenic tobacco cells (Figure 3). Together, these results confirmed that constituent proteins are functional upon release from the IntF2A‐based polyprotein precursor.

**Figure 4:**
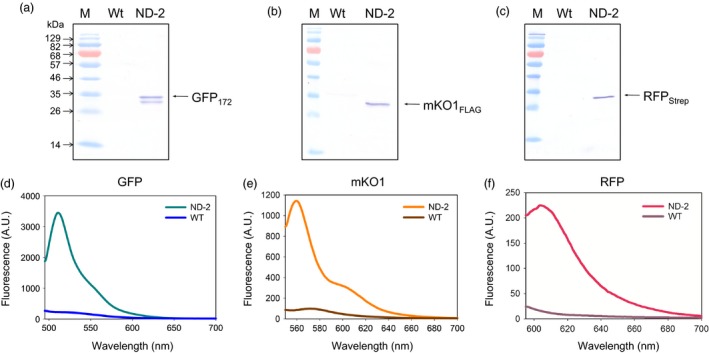
Co‐expression of three POIs using the IntF2A‐based polyprotein vector system. Efficient *in vivo* processing of three POIs was confirmed by Western blots of transgenic NT1 cell extracts expressing ND‐2 using anti‐GFP (a), anti‐mKO1 (b) and anti‐Strep Tag (c) antibodies, respectively. Processed POIs in ND‐2 show characteristic fluorescence spectra (d–f), which are distinctive from the background autofluorescence in untransformed wild‐type controls (WT). POIs, proteins of interest.

As modification of cellular pathways in plants often requires manipulation of multiple enzymes simultaneously, capability of the IntF2A system for co‐expressing more than two POIs was investigated. Here co‐expression of three proteins, that is GFP_172_, monomeric Kusabira‐Orange 1 fluorescent protein with a FLAG Tag at the C‐terminus (mKO1_FLAG_), and RFP_Strep_, using the IntF2A polyprotein system, was examined in tobacco NT1 cells (ND‐2; Figure 1). Essentially, complete release of all three proteins was confirmed by Western blots probed with anti‐GFP, FLAG Tag and Strep Tag antibodies, respectively (Figure 4a–c). Similar to the observation in the two‐protein co‐expression construct, ND‐1, all processed fluorescent reporters from ND‐2 were verified to be functional based on fluorescence spectroscopy (Figure 4d–f).

**Figure 5:**
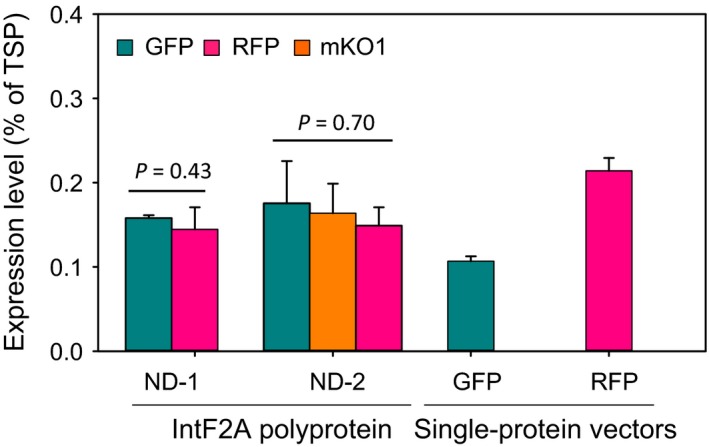
Comparing expression levels of POIs (GFP, mKO1 and RFP) from IntF2A‐based polyprotein vectors vs. single‐protein vectors in transgenic tobacco NT1 cells. To estimate the POI expression levels, fluorescence intensities of individual POIs in the transgenic cell extracts were corrected for background autofluorescence, by subtracting fluorescence of wild‐type extract prepared with the same total soluble protein concentration, followed by conversion to protein concentrations using a calibration curve. Data represent the mean of three transgenic callus replicates ±SD. The high *P*‐values from one‐way ANOVA indicated that the constituent POI expression levels in the same construct are not significantly different for either ND‐1 or ND‐2. TSP, total soluble proteins; POIs, proteins of interest.

In the IntF2A‐based polyprotein system, constituent POIs were translated from a single transcript; thus, it is possible that stoichiometric protein co‐expression can be achieved by the system. Figure 5 shows that processed GFP and RFP from ND‐1 accumulated to similar levels inside the cells. Similarly, all three proteins expressed from ND‐2 accumulated to a similar level (Figure 5). In addition, co‐expression using the IntF2A polyprotein system does not compromise expression levels when compared with those using single‐protein vectors, as shown in Figure 5 (in single‐protein vectors, GFP_172_ or RFP_Strep_ alone was expressed from the (ocs)_3_/mas promoter).

### Determination of processing sites within the IntF2A‐based polyprotein

**Figure 6:**
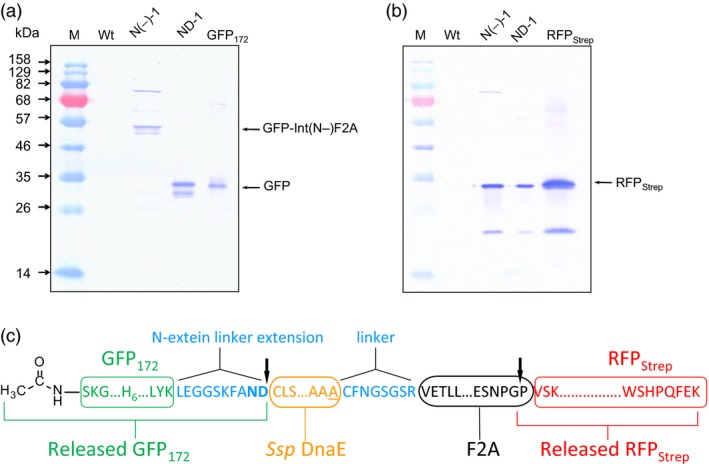
Intein and F2A are responsible for autocatalytic release of the upstream and downstream POIs, respectively. (a) GFP Western blot of ND‐1 and N(‐)‐1 cell extracts confirms that the intein domain is solely responsible for release of the upstream POI, GFP_172_. (b) RFP Western blot shows that inactive N‐terminal intein cleavage does not impair C‐terminal cleavage mediated by F2A in N(‐)‐1. (c) *In vivo* cleavage of the ND‐1 polyprotein precursor occurred at the expected sites (indicated by the arrows), as confirmed using N‐terminal amino acid sequencing and ESI‐TOFMS analysis of released POIs. POIs, proteins of interest.

To investigate whether the observed cellular processing of polyprotein precursors indeed resulted from autoprocessing activity of the IntF2A fusion domain, N‐terminal cleavage activity of the IntF2A domain was blocked by introducing a C1A mutation in the intein sequence (N(‐)‐1 in Figure 1). In addition, the intein sequence was flanked by amino acid residues Leu‐Glu‐Tyr and Gly‐Gly‐Ser‐Arg at the N‐ and C‐terminal junctions to further diminish its autocleavage activity. As shown in Figure 6a,b, release of POI1 was impaired by these mutations, whereas efficient release of the POI2 was still preserved. This result confirmed that the N‐terminal cleavage activity of the *Ssp* DnaE intein domain was solely responsible for release of the POI1.

The exact processing sites within the IntF2A polyprotein were determined by N‐terminal sequencing and ESI‐TOFMS of the processed POIs purified from the extract of transgenic NT1 cells expressing ND‐1. N‐terminal amino acid sequencing indicated that the released RFP_Strep_ (POI2) has a proline residue directly upstream of its native N‐terminal sequence (VSKGEE) (Figure 6c). This result is consistent with the known mechanism of F2A‐mediated processing by which peptide bond formation between the last two amino acid residues, glycine and proline, is disrupted during translation. Regarding the POI1, the molecular mass measured using ESI‐TOFMS (28 590 Da) matches that of an N‐terminal acetylated GFP_172_ plus the C‐terminal N‐extein linker (LEGGSKFAND) (calculated mass 28 593 Da) (Figure 6c). Note that after the initiator methionine was removed from GFP_172_ by methionine aminopeptidase, serine at the N‐terminus, being the most common substrate for N‐terminal acetyltransferase, is likely to be acetylated. The notion of post‐translational modification at the N‐terminus of POI1 is in agreement with our N‐terminal sequencing result which indicated that the N‐terminus was blocked. This result is also consistent with our previous findings of a dual‐intein polyprotein expression system when used in plants (Zhang *et al*., 2015). Collectively, our results support that the observed cellular processing of the IntF2A‐based polyprotein was indeed mediated by the specific actions of intein and F2A sequences.

### Subcellular targeting of proteins processed from the IntF2A‐based polyprotein

**Figure 7:**
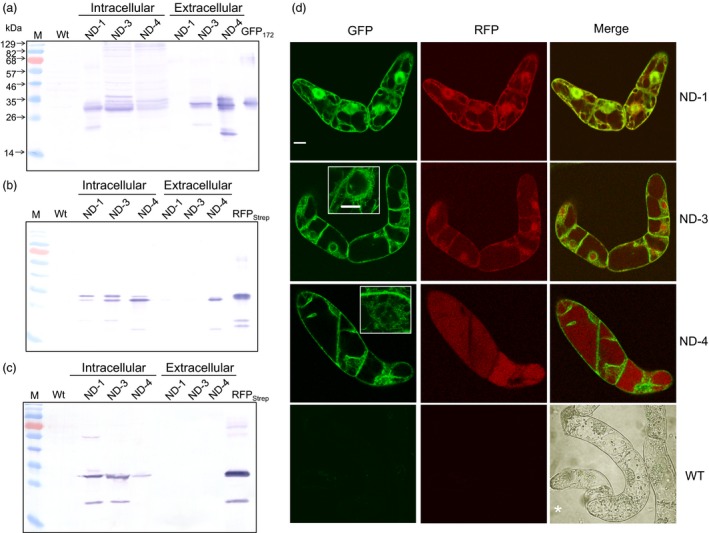
Subcellular targeting of constituent POIs using IntF2A‐based polyprotein vectors. (a–c) Secretion of GFP and RFP proteins released from the IntF2A polyprotein precursors (ND‐1, ND‐3 or ND‐4) was probed using Western blots with anti‐GFP antibody (a), anti‐RFP antibody (b) or anti‐Strep Tag antibody (c). Confocal fluorescence microscopy of transgenic tobacco NT1 cells confirmed proper cellular targeting of the released POIs (d). Insets indicate the GFP‐labelled ER network in the cells. Scale bar: 10 μm. *Transmitted light image was included to indicate the WT cell boundary. POIs, proteins of interest.

To examine whether proteins released from the IntF2A‐based polyprotein precursor can be independently targeted to different cellular compartments, endoplasmic reticulum (ER) targeting signal was integrated upstream of either only POI1 (ND‐3) or both POI1 and POI2 (ND‐4) in the IntF2A polyprotein cassette. The *Arabidopsis thaliana* basic chitinase signal peptide (SP1) and the rice α‐amylase signal peptide (SP2) were used to direct ER targeting of POI1 (GFP_172_) and POI2 (RFP_Strep_), respectively, as depicted in Figure 1. For both ND‐3 and ND‐4 cassettes, GFP_172_ was effectively released from the polyprotein precursor (Figure 7a, intracellular lanes), indicating active intein autocleavage in the ER. Moreover, GFP_172_ was detected in the spent media of both ND‐3 and ND‐4 suspension cultures, but not in the ND‐1 culture media (Figure 7a, extracellular lanes). Effective release of RFP_Strep_ with both ND‐3 and ND‐4 was also observed (Figure 7b, intracellular lanes) and, as anticipated, extracellular RFP was detected only in the ND‐4 culture media (Figure 7b, extracellular lanes). The presence of a proline residue at the N‐terminus of the SP2, as a result of F2A action, did not impair the ability of signal peptide to target the RFP_Strep_ to ER, and inclusion of SP2 was necessary to direct the RFP for secretion since no extracellular RFP was found with ND‐3. It was also noted that the C‐terminal Strep Tag was removed from the RFP_Strep_ in the ND‐4 expressing cells (Figure 7c).

Differential subcellular localization of the processed POIs was further examined using confocal laser scanning microscopy (Figure 7d). For the cytosolic ND‐1 cassette, both GFP_172_ and RFP_Strep_ localized to the cytosol as well as the nucleus. For small cytosolic proteins such as GFP and RFP, it is well known that they may translocate to the nucleus on their own via nonspecific diffusion across the nuclear pores (Seibel *et al*., 2007). However, when a signal peptide was appended to the GFP as in ND‐3 and ND‐4, the processed GFP_172_ was found localized to the ER network as well as the cell envelope, but not in the nucleus. Subcellular distribution of RFP fluorescence within the ND‐3 cells is similar to that of ND‐1. Because the translational recoding event mediated by F2A occurs cotranslationally, the presence of SP1 alone (without SP2) in ND‐3 does not allow the protein downstream of F2A to enter the ER. For ND‐4, while we detected extracellular RFP in the spent media (Figure 7b), RFP fluorescence was also found in the vacuole (Figure 7d).

As many nonstructured C‐terminal peptide sequences have been reported to direct vacuole targeting in plants (Xiang *et al*., 2013), it may explain the observed vacuole‐targeting property of the C‐terminal F2A extension. To this end, another notable observation from this study is that some of the RFP_Strep_ released from the ND‐4 polyprotein accumulated inside the vacuole (Figure 7d, ND‐4) although we did also detect extracellular RFP (Figure 7b). In an earlier study of plant membrane traffic using polyproteins (Samalova *et al*., 2006), secretion of a monomeric RFP (a DsRed variant (Campbell *et al*., 2002) very similar to the mCherry RFP used in the present study) into the apoplastic space of *N. benthamiana* leaf was noted when an N‐terminal secretory signal peptide was incorporated. Therefore, the C‐terminal Strep Tag appended to the RFP in ND‐4 may be recognized as a putative vacuole sorting determinant. The Strep Tag sequence on some of the processed RFP_Strep_ might have been degraded along the secretory pathway and avoided recognition by the vacuole sorting receptor and, as a result, was secreted into the media. To this end, we detected a RFP band on Western blot of extracellular ND‐4 samples using an anti‐mCherry antibody (Figure 7b, lane 5), but not with an anti‐Strep Tag antibody (Figure 7c, lane 5). Evidence from our prior study indicated very active peptidase activity in the secretory system of the tobacco cells that leads to digestion of nonstructured terminal peptide linker extensions (Zhang *et al*., 2011). For the RFP subpopulations with the intact Strep Tag, once translocated into the plant vacuole, the tag sequence might also be removed. When probed using an anti‐mCherry antibody, the band on the Western blot corresponding to RFP in the intracellular extract for ND‐4 is smaller than that of ND‐1 and ND‐3 (Figure 7b, lanes 3–5). Furthermore, the same RFP product is not visible when probed with an anti‐Strep Tag antibody (Figure 7c, lane 5).

### Processing of the IntF2A‐based polyprotein without N‐extein linker extension

**Figure 8:**
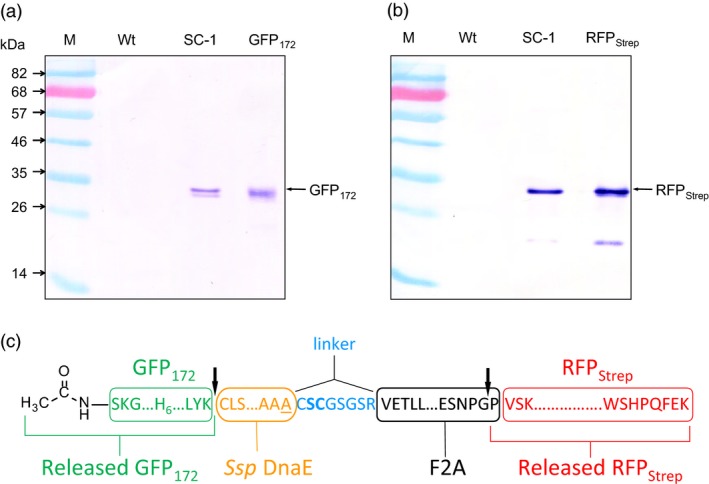
Efficient processing of the IntF2A‐based polyprotein can be achieved without N‐extein extension (SC‐1). (a,b) Processing of the SC‐1 polyprotein without N‐extein linker extension (cf. ND‐1) in NT1 cells analysed using Western blots probed with anti‐GFP (a) and anti‐Strep Tag (b) antibodies, respectively. (c) N‐terminal amino acid sequencing and ESI‐TOFMS analysis confirmed that *in vivo* cleavage of the IntF2A polyprotein without N‐extein linker extension occurred at the expected sites (indicated by the arrows).

The IntF2A‐based polyprotein system described above resulted in release of POI1 with residual amino acids from the N‐extein linker. While proper function of the processed POI1, that is GFP_172_, was preserved despite addition of this non‐native sequence at its C‐terminus, other proteins might not tolerate this extension. A previous study (Amitai *et al*., 2009) reported that intein N‐terminal autocleavage can be accelerated by modifying amino acid residues proximal to its N‐ or C‐terminal junctions (cf. Figure 1). Hence, we modified the ND‐1 polyprotein cassette by removing the N‐extein linker extension and substituting the C+2 and C+3 residues with N‐terminal cleavage enhancers, serine and cysteine, respectively (Amitai *et al*., 2009). Efficient processing of this modified IntF2A‐based polyprotein (SC‐1; Figure 1) was demonstrated in transgenic tobacco NT1 cells using Western blots probed with both anti‐GFP and Strep Tag antibodies (Figure 8a,b). The results suggested that elimination of the N‐extein linker did not attenuate processing efficiency of the IntF2A domain. N‐terminal amino acid sequencing together with ESI‐TOFMS analysis (spectra not shown) on processed proteins revealed that processing of the SC‐1 polyprotein occurred at the expected sites, and the resulting processed upstream GFP_172_ preserved its native C‐terminus (Figure 8c).

### Production of a functional IgG antibody using the IntF2A‐based polyprotein system

**Figure 9:**
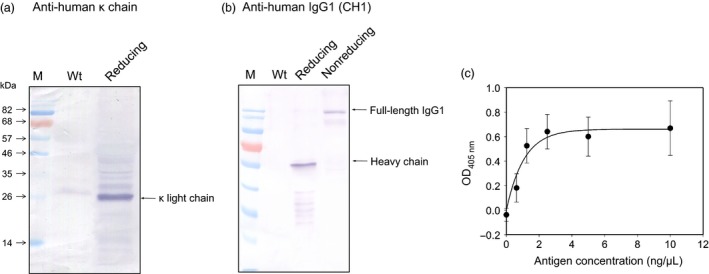
Expression and assembly of chimeric anti‐His Tag IgG1 in plants using the IntF2A‐based polyprotein vector system. (a,b) Demonstration of *in vivo* processing and assembly of anti‐His Tag antibody complex based on Western blot analysis. Total soluble protein from *Nicotiana benthamiana* leaf extract was hybridized with anti‐human κ light chain antibody (a) or anti‐human IgG1 antibody (b). (c) Biological activity of the plant‐made anti‐His Tag antibody was measured based on sandwich ELISA assay against antigen GFP_H__is_. Error bars indicate standard deviation of the mean from triplicate samples.

To evaluate the applicability of the optimized IntF2A‐based polyprotein system for expression of useful multimeric protein complexes, a chimeric anti‐His Tag antibody was chosen as a model. To construct this chimeric IgG antibody, we combined the variable domains of a murine anti‐His Tag ScFv antibody (Kaufmann *et al*., 2002) with the constant domains of human IgG1 (Dodev *et al*., 2014). The antibody expression cassette (Figure 1, SC‐2) was assembled by flanking the optimized IntF2A autoprocessing domain (without the N‐extein linker extension) with the chimeric kappa light chain (as POI1) and gamma heavy chain (as POI2) of the anti‐His Tag antibody. The assembled SC‐2 was transiently expressed in *N. benthamiana* leaves *via* agroinfiltration. The antibody product was analysed 4 days postinfiltration. Processing of the anti‐His Tag antibody expressed in *N. benthamiana* was characterized using Western blot probed with anti‐human kappa light chain and anti‐human IgG antibodies that recognize the constant region of the light chain and heavy chain of human IgG, respectively, under reducing or nonreducing condition. Both light chain and heavy chain fragments of the anti‐His Tag antibody were fully liberated from the polyprotein precursor under reducing condition (Figure 9a,b). Detection of the heterodimeric antibody complex under nonreducing condition indicated that assembly of the full‐length anti‐His Tag antibody was successfully established via intermolecular disulphide bonds between released light chain and heavy chain (Figure 9b, Lane 4).

Biological activity of the antibody expressed from SC‐2 in *N. benthamiana* was evaluated using a sandwiched ELISA based on the antibody's binding affinity towards the C‐terminal hexa‐histidine tagged GFP (GFP_His_). As shown in Figure 9c, the anti‐His Tag antibody expressed from the SC‐2 polyprotein exerted proper antigen binding activities in a dose‐dependent manner. This result, together with Western blot analysis, confirmed that fully assembled bioactive antibody complex was successfully produced from the IntF2A‐based polyprotein system. With the N‐extein linker‐free IntF2A system, the processed antibody light chain preserved its authentic C‐terminus without addition of any residual amino acids, which is superior to previously reported approaches (Fang *et al*., 2005). The native N‐terminus of the processed downstream heavy chain fragment should also be preserved as the proline residue introduced by the action of F2A is removed upon signal peptide cleavage.

## Discussion

In this study, we developed an IntF2A fusion protein domain with hyperactive autoprocessing activity to direct coordinated co‐expression of multiple proteins in plants from a single open reading frame encoding a polyprotein precursor. This system has important advantages over the polyprotein expression system that is based on F2A alone. While F2A sequences from 18 to 58 amino acid residues have been used, it is well known that the shorter versions of F2A give lower translational recoding efficiencies. On the other hand, when using longer versions of F2A, the F2A extension that remains on the POI's C‐terminus could be highly problematic (Donnelly *et al*., 2001a; Minskaia *et al*., 2013). In the IntF2A system reported here, the long 58aa version of F2A is utilized to maximize the efficiency in releasing its downstream protein, but it is removed swiftly by the action of the intein domain to eliminate potential negative effects imposed by the long F2A overhang. We have succeeded in co‐expressing as many as three POIs from a single IntF2A‐based polyprotein. Efficient cellular processing of the IntF2A‐based polyproteins was demonstrated in cultured tobacco NT1 cells, in different organs of transgenic *N. tabacum* plants, as well as in lettuce and *N. benthamiana*. POIs released from the polyprotein precursors displayed proper function and accumulated to similar levels. N‐terminal amino acid sequencing together with ESI‐TOFMS analysis revealed that processing of the IntF2A‐based polyprotein is consistent with the known protein processing mechanism mediated by intein and the F2A peptide. By optimizing the C‐extein linker residues within the IntF2A domain, released POIs can preserve its native C‐terminus. Furthermore, we demonstrated important practical applications of the IntF2A technology beyond just proof of concepts with reporters, by successfully producing a correctly assembled and biologically functional IgG antibody molecule. Mass production of pharmaceutically relevant proteins such as antibodies in plants through molecular farming, especially with transient expression systems, has drawn increasing interests due to its simplicity and low‐cost nature compared with the conventional microbial and mammalian cell culture production methods (Arntzen, 2015; Ma *et al*., 2003).

Processing of the IntF2A‐based polyprotein is initiated during protein translation, in which peptide bond formation between the last two amino acids of the F2A peptide, that is glycine and proline, is disrupted by the unique translational recoding activity of the F2A sequence (Donnelly *et al*., 2001b). It has been hypothesized that the translational recoding event requires specific interaction of the ribosome exit tunnel with the nascent 2A peptide to constrain the conformational space of the peptidyl(2A)‐tRNA^gly^ ester bond in the ribosome P‐site to ‘jam’ further elongation (Minskaia *et al*., 2013; Roulston *et al*., 2016; Yan *et al*., 2010). Since the ribosome exit tunnel can accommodate 30–40 amino acids, the activity of F2A sequences with shorter than 30 residues may be influenced by the C‐terminal sequence of the protein upstream (Minskaia and Ryan, 2013). Indeed, it is known that improved translational recoding efficiency can be achieved by employing longer versions of F2A which incorporate additional residues derived from the native viral sequence upstream of the core F2A sequence (Donnelly *et al*., 2001a; Minskaia *et al*., 2013). The long C‐terminal F2A extension could, however, negatively impact protein conformation, protein trafficking, as well as post‐translational modifications and protein activities that require native carboxyl terminus (Minskaia *et al*., 2013). Using the InF2A domain, this adverse effect is overcome by virtue of the N‐terminal autocleavage activity of the intein mutant within the IntF2A fusion domain. While N‐terminal intein cleavage is considered a post‐translational process, previous kinetic studies revealed that N‐terminal intein cleavage rates determined *in vitro* are much faster than the average protein synthesis rates in plant cells (Amitai *et al*., 2009; Li *et al*., 2012; Martin *et al*., 2001; Saleh *et al*., 2011; Trewavas, 1972). By incorporating N‐terminal cleavage accelerating residues in the flanking extein regions, for example Asn‐Asp at intein N‐terminal junction or Cys‐Ser‐Cys bordering the intein C‐terminus, cleavage rate was found to increase by fourfold compared to that with the native flanking extein residues (Amitai *et al*., 2009). Therefore, it is reasonable to assert that release of POI1 from the polyprotein precursor occurs immediately after the intein is folded to allow rapid removal of the IntF2A domain from the C‐terminus of POI1 to avoid potential adverse effects inside the cell.

As an exemplified case where the presence of F2A at the C‐terminus of a cytosolic protein may negatively impact the protein, we observed that when GFP_172_ and RFP_Strep_ were separated by the 58aa F2A sequence but without the intein domain (i.e. forming a GFP_172_‐F2A‐RFP_Strep_ polyprotein termed ‘2A‐1’), the amount of GFP_172_‐F2A detected was much lower than that of RFP_Strep_ (Figure S2a). In addition to fluorescence‐based measurement, the observed low GFP/RFP ratio was confirmed by scanning densitometry analysis of Western blots (data not shown). Interestingly, the GFP_172_‐F2A fragment appeared smaller than the GFP_172_ standard (Figure S2b). These observations suggested that although the C‐terminal F2A extension does not hamper the fluorescence function of GFP_172_, it might have triggered destabilization of GFP_172_, resulting in less protein accumulated and with a lower molecular mass. This problem can be circumvented with the IntF2A approach in which the intein domain enables rapid removal of the C‐terminal F2A extension to achieve more balanced production of the constituent POIs (Figure S2a, ND‐1 and SC‐1).

Correct differential subcellular targeting of the POIs derived from IntF2A polyproteins to cytosol and the secretory system was demonstrated in this study, suggesting the IntF2A approach does not interfere with protein trafficking in plant cells. In the event the C‐terminal F2A sequence remained attached to an ER‐targeted protein, it might cause erroneous protein sorting (François *et al*., 2004; Samalova *et al*., 2006). To this end, we investigated tobacco NT‐1 cells expressing the N(‐)‐2 polyprotein (Figure 1) that consists of a secretory GFP_172_ and a cytosolic RFP_Strep_, separated by a mutated IntF2A with abolished N‐terminal autocleavage activity, that is Int(N‐)F2A (containing a C1A mutation in the intein). As expected, we detected GFP_172_‐Int(N‐)F2A in the cell extract indicating RFP_Strep_ was released from the N(‐)‐2 polyprotein (since F2A was active) but GFP_172_ was not (Figure S3a). In the cell extract, we also detected protein species smaller than that of the GFP_172_ standard but cross‐reacted with the GFP antibody on the Western blot (Figure S3a). However, in the spent culture media, no protein products were detected on anti‐GFP Western blot for the N(‐)‐2 cells (Figure S3b). It is plausible that the lack of detectable extracellular GFP_172_‐Int(N‐)F2A implied that protein mistargeting might have occurred along the secretory pathway. This notion is substantiated by the observation of strong GFP fluorescence localized to the vacuole of the tobacco NT1 cells expressing N(‐)‐2 under confocal fluorescence microscopy examination (Figure S3c). The vacuole targeted GFP_172_‐Int(N‐)F2A was partially degraded, probably due to hydrolysis by proteinases that reside in the secretory pathway or the vacuole, as multiple protein bands were detected by Western blot analysis (Figure S3a). The undesired vacuole sorting mediated by the F2A sequence has been reported in earlier studies that the secretory protein anterior to the F2A peptide is prone to accumulation in the vacuole (François *et al*., 2004; Samalova *et al*., 2006). Conversely, IntF2A with an active intein (e.g. in ND‐3 or ND‐4) directs efficient protein secretion as shown in Figures 7a and S3b. This again highlights the importance of rapid removal of the C‐terminal F2A extension to avoid potential adverse effects, for both ER‐targeted and cytosolic proteins. The successful expression and secretion of GFP_172_ from ND‐3 and ND‐4 also indicated that the intein autocleavage activity is insensitive to the cellular oxidative environment, since the ER lumen is more oxidative than that of the cytoplasm, and this finding is in agreement with our previous observation (Zhang *et al*., 2015).

In comparison with the dual‐intein‐based polyprotein expression system recently reported by our group (Zhang *et al*., 2015), in which a pair of self‐excising mini‐intein variants (having N‐ and C‐terminal autocleavage activity, respectively) fused in tandem, the IntF2A approach has a number of important advantages. These include (i) smaller molecular size of the IntF2A domain, (ii) cotranslational cleavage mediated by the F2A peptide, (iii) adding only a single proline residue to the POI trailing the autoprocessing domain (in case of a secretory POI, the proline residue will be removed along with the signal peptide by the signal peptidase and hence the POI can preserve its authentic N‐terminus) and (iv) higher efficiency in releasing the downstream POI and hence more balanced co‐expression among the POIs in plants. The utility of the IntF2A approach can also be further extended, for instance, by synergistic integration with the bidirectional promoter systems (Kumar *et al*., 2015) to further increase the number of transgenes that can be co‐expressed in plants.

In summary, the IntF2A‐based polyprotein system enables highly efficient coordinated co‐expression of multiple proteins from a single transgene in plant cells and whole plants. Its many unique advantages as described throughout this report make the IntF2A a highly powerful molecular tool for plant sciences and biotechnology.

## Experimental procedures

### Design and assembly of genetic constructs

For the ND series of cassettes, asparagine and aspartate as flanking N‐extein amino acids have been reported to accelerate N‐terminal intein cleavage (Amitai *et al*., 2009). The C‐extein linker located between the intein and the F2A sequence consists of three native C‐extein residues (Cys‐Phe‐Asn) followed by Gly‐Ser‐Gly‐Ser‐Arg. For the SC cassettes, the N‐extein linker was completely eliminated to preserve the C‐terminus of POI1. To enhance the N‐terminal cleavage efficiency, the C‐extein linker was modified by substituting the native C+2 and C+3 residues phenylalanine and asparagine with serine and cysteine, respectively (Amitai *et al*., 2009). Fluorescent proteins GFP_172_ (a GFP variant with a hexa‐histidine sequence inserted between amino acids 172 and 173) (Paramban *et al*., 2004) and RFP_Strep_ (mCherry with a C‐terminal Strep Tag) were used as reporters in most polyprotein cassettes (as POI1 and POI2, respectively) for facile detection of protein processing.

Detailed experimental procedures for vector construction,nucleic acid sequence of the IntF2A domain, along with a list of primers used in this study (Appendix S1; Table S1), can be found in the supporting information.

### Plant transformation and protein extraction

The pE1775 vectors containing IntF2A‐based polyprotein sequences were transformed into *Agrobacterium tumefaciens* C58C1 via electroporation. Stable transformation of tobacco NT1 cells and *N. tabacum* plants was performed using *Agrobacterium* cocultivation approach as described in previous publications (Fisher and Guiltinan, 1995; Mayo *et al*., 2006). Hygromycin was used for transformation selection. Fluorescence and Western blot analysis were used to screen the highest expression lines which were selected for subsequent characterization. Vacuum‐assisted agroinfiltration for transient expression in leaf tissues of *N. benthamiana* and Romaine lettuce was performed as described previously (Zhang *et al*., 2011).

Total soluble intracellular proteins were extracted in boric acid extraction buffer following a procedure described previously (Peckham *et al*., 2006). To collect secreted proteins, apoplastic fluid or spent media of transgenic NT1 calli or suspension cultures, respectively, were filtered through a 10‐μm nylon mesh filter. The crude filtrates were clarified by filtering through a Whatman #1 filter paper. Total protein concentration was determined using Bradford protein assay (Bradford, 1976) (Bio‐Rad, Hercules, CA) and target proteins were tracked by fluorescence measurement using a Hitachi F‐2500 fluorescence spectrophotometer (Hitachi High Technologies America, Pleasanton, CA).

### SDS‐PAGE and Western blot

Protein extracts were mixed with SDS‐PAGE sample buffer and denatured at 95°C for 5 min under reducing or nonreducing conditions. After brief centrifugation, denatured protein samples were subjected to 12% SDS‐PAGE and blotted onto PVDF membranes. Rabbit anti‐GFP (Invitrogen, Grand Island, NY) was used to detect GFP_172_, while RFP_Strep_ was detected by rabbit anti‐Strep Tag antibody (Genscript, Piscataway, NJ) or rabbit anti‐RFP antibody (Biovision, Milpitas, CA). Rabbit anti‐human κ light chain and rabbit anti‐human IgG antibodies (Abcam, Cambridge, MA) were used to detect light and heavy chains of anti‐His Tag antibody, respectively. Alkaline phosphatase‐conjugated goat anti‐rabbit antibody (Southern Biotech, Birmingham, AL) was used as the secondary antibody. Immunoreactive bands were visualized by NBT/BCIP coupled chromogenic reaction. The GFP_172_ and RFP_Strep_ protein standards were expressed in *Escherichia coli* BL21 (DE3) and purified using affinity column chromatography as described previously (Zhang *et al*., 2015).

### Fluorescence confocal microscopy

Subcellular sorting of processed proteins was analysed using an Olympus Fluoview FV‐1000 confocal laser scanning microscope system mounted on an Olympus IX‐81 inverted microscope (Nikon, Tokyo, Japan) and performed at the Biological Electron Microscopy Facility at the University of Hawaii. Cell images were observed with a UPLSAPO 20× lens [numerical apertures (NA), 0.70; Nikon, Tokyo, Japan], along with 3X digital zooming. Cells were excited with laser beams at 488 and 543 nm, respectively, for detection of GFP and RFP (mCherry) fluorescence. Filters BA505‐525 and BA560IF were used for collecting fluorescence emission of GFP and RFP, respectively.

### Protein purification for N‐terminal amino acid sequencing and ESI‐TOFMS analysis

Processed RFP_Strep_ was purified from ND‐1 and SC‐1 cell extracts using Strep‐Tactin chromatography (Qiagen, Hilden, Germany) according to the procedure published previously (Zhang *et al*., 2015). Unbound flow‐through from the Strep‐Tactin column was subject to hydrophobic interaction chromatography, followed by immobilized metal affinity chromatography (GE Healthcare, Marlborough, MA) for purification of processed GFP_172_ as described previously (Peckham *et al*., 2006; Zhang *et al*., 2015). Purified GFP_172_ and RFP_Strep_ were further processed using SDS‐PAGE and blotted onto PVDF membrane. Target protein bands were excised for N‐terminal amino acid sequencing using Edman degradation approach (performed by the Protein Facility at Iowa State University). ESI‐TOFMS analysis of purified processed GFP_172_ protein was carried out as described previously (Zhang *et al*., 2015).

### ELISA assay for chimeric antibody against hexa‐histidine tagged proteins

Biological activity of plant expressed anti‐His Tag antibody was assayed using sandwich ELISA. Protein extract of agroinfiltrated *N. benthamiana* leaves was coated in triplicate onto a 96‐well plate. After blocking with 1% BSA (in PBS with 0.05% Tween 20), different concentrations (0, 0.625, 1.25, 2.5, 5 and 10 μg/mL) of C‐terminal hexa‐histidine tagged GFP (GFP_His_) were added. Captured GFP_His_ was detected using rabbit anti‐GFP antibody (Cell Sciences, Canton, MA), followed by alkaline phosphatase‐conjugated goat anti‐rabbit antibody. ELISA signal was generated by incubating with chromogenic substrate *p*‐nitrophenyl phosphate and measured by absorbance at 405 nm. Data presented in Figure 9 were normalized by subtracting the background generated from the wild‐type leaf extract hybridizing with corresponding antigen concentrations.

## Conflict of interest

B.Z. and W.W.S. are the inventors of a US patent for the autoprocessing domain technology.

## Supporting information


**Figure S1** Cellular processing of the IntF2A based polyprotein.
**Figure S2** Presence of F2A at the C‐terminus of a cytosolic protein may negatively impact the protein.
**Figure S3** C‐Terminal F2A extension causes mis‐targeting of POI to the vacuole when an ER targeting signal is included at the N‐terminus of the POI.
**Table S1** List of primers used in this study.
**Appendix S1** Supplementary experimental procedures and nucleic acid sequence of the IntF2A domain.
