# Supplementary material for: Coordinated protein co‐expression in plants by harnessing the synergy between an intein and a viral 2A peptide
Source: Plant Biotechnol J. 2017 Mar 30;15(6):718–28. doi: 10.1111/pbi.12670 (PMC5425387; doi:10.1111/pbi.12670)
Supplement: Supplementary file 1 — Figure S1 Cellular processing of the IntF2A based polyprotein. Figure S2 Presence of F2A at the C‐terminus of a cytosolic protein may negatively impact the protein. Figure S3 C‐Terminal F2A extension causes mis‐targeting of POI to the vacuole when an ER targeting signal is included at the N‐terminus of the POI. Table S1 List of primers used in this study. Appendix S1 Supplementary experimental procedures and nucleic acid sequence of the IntF2A domain. [file PBI-15-718-s001.docx]

**SUPPORTING INFORMATION**

**Coordinated Protein Co-expression in Plants by Harnessing the Synergy between an Intein and a Viral 2A Peptide**

Bei Zhang^1^, Madhusudhan Rapolu^1^, Sandeep Kumar^2^, Manju Gupta^2^, Zhibin Liang^1^, Zhenlin Han^1^, Philip Williams^3^, and Wei Wen Su^1*^

1. Department of Molecular Biosciences and Bioengineering, University of Hawaii at Manoa, Honolulu, Hawaii 96822, USA
2. Dow AgroSciences LLC, Indianapolis, Indiana 46268, USA
3. Department of Chemistry, University of Hawaii at Manoa, Honolulu, Hawaii 96822, USA

* To whom correspondence should be addressed: Tel: 1-808-956-3531

**Figure S1.** Cellular processing of the IntF2A based polyprotein.

**Figure S2.** Presence of F2A at the C-terminus of a cytosolic protein may negatively impact the protein.

**Figure S3.** C-Terminal F2A extension causes mis-targeting of POI to the vacuole when an ER targeting signal is included at the N-terminus of the POI.

**Table S1.** List of primers used in this study.

**Supplementary experimental procedures:** Vector construction

**Nucleic acid sequence of the IntF2A domain**

**References cited**

**Figure S1.** Cellular processing of the IntF2A based polyprotein. (ocs)_3_/mas promoter and agrocinopine synthase (*ags*) terminator (Lee et al. 2007) were used in all constructs reported in this study.

*

**Figure S2.** Presence of F2A at the C-terminus of a cytosolic protein may negatively impact the protein. (a) Uneven accumulation of POIs processed from the GFP_172_-F2A-RFP polyprotein (2A-1; lacking the intein domain) was noted, while balanced co-expression of the POIs was noted using IntF2A vectors (ND-1 and SC-1). To estimate the POI expression levels, fluorescence intensities of the individual POIs in the transgenic cell extracts were corrected for background autofluorescence, by subtracting fluorescence of WT extract prepared with the same total soluble protein concentration, followed by conversion to protein molar concentrations (C_GFP_ and C_RFP_) using calibration curves. Data represent the mean of three transgenic callus replicates ± SD. Statistical significance of the differences between mean expression levels of POIs for a given construct was determined using one-way ANOVA and the resulting p-values are shown. Asterisk denotes significant differences between C_GFP_ and C_RFP_ in 2A-1. (b) The GFP_172_-F2A fragment released from the 2A-1 polyprotein appeared smaller than GFP_172_ on the western blot probed with anti-GFP antibody (cf. Figs. 2a and 8a), suggesting potential proteolytic digestion prompted by the C-terminal F2A extension.

**Figure S3.** C-Terminal F2A extension causes mis-targeting of POI to the vacuole when an ER targeting signal is included at the N-terminus of the POI. NT1 cells expressing N(-)-2 that contains an inactive intein, i.e. Int(N-), in the IntF2A domain were analyzed. (a) Western blot of N(-)-2 cell extract probed with anti-GFP antibody revealed GFP_172_-Int(N-)F2A and its degradation products. (b) 3 g (fresh weight) of ND-3 or N(-)-2 calli were suspended in 3 ml of MS media (Murashige and Skoog, 1962) for 2 days and spent media analyzed using western blot probed with anti-GFP antibody. No GFP was detected in the spent medium of NT1 cells expressing N(-)-2. (c) Confocal fluorescence microscopy images of the NT1 cells expressing N(-)-2 show that GFP fluorescence is mainly localized in the vacuole (cf. ND-3 in Fig. 7d).

**Table S1.** List of primers used in this study.

| Name | | Sequence (5’-3’) | |
| --- | --- | --- | --- |
| *Sal*I-mGFP5 | | ACGCGTCGACCAAGGAGATATAACAATGAGTAAAGGAGAAGAACT | |
| GFP-*Xho*I-R | | TGCCTCGAGGTGATGGTGATGGTGATGC | |
| GFP-GS-*Xba*I-R | | AACTCTAGAACCAGAACCTTTGTATAGTTCATCCATGCC | |
| mCherry-2A-F | | GAGACGTCGAGTCCAACCCTGGGCCCGTGAGCAAGGGCGAGGAGGATAACATG | |
| mCherry-STREP-R | | CAAACTGAGGATGTGACCATCCAGAACCCTTGTACAGCTCGTCCATGCCGCCG | |
| GKZ-R | | CTGGAGCTCTCATTTTTCAAACTGAGGATGTGAC | |
| mKO1-*Apa*I-F | | GCATGGGCCCGTGAGTGTGATTAAACCAGAGATGAAGA | |
| mKO1-R | | CATCATCCTTATAATCTCCAGAACCGGAATGAGCTACTGCATCTTCTACCAGC | |
| FLAG-*Sac*I-R1 | | TGCATGAGCTCCTTATCATCATCATCCTTATAATCTCCAGAAC | |
| G11c-*Sac*I-DnaE-F | | GTAACTGCTGCTGAGCTCGGAGGATCTAAGTTTGCAAATG | |
| M13F  mCherry-SphI-F  Sal5er-F | | GTAAAACGACGGCCAG  CAGCCGGCATGCTGAGCAAGGGCGAGGAG  AGTCGACCAAGGAGATATAACAATG | |
| mGFP5-His-*Xho*I-R | | AGTACTCGAGGTGATGGTGATGGTGATGACTTCCAATCCCAGCAGCTGTTACA | |
| mGFPS2E | | GCCGAATTCAGTAAAGGAGAAGAACTTTTC | |
| DnaE-*Xho*I-F-2 | | ACTCCTCGAGTATGCATTGTCCTTCGGAACTGAGATACTTAC | |
| DnaE-*Xba*I-R-2 | | GTATTCTAGAACCACCAGCTGCAGCAATAGCACCGTTTGCGAG | |
| G172-DnaE-F | | ACTATACAAATGTTTGTCCTTCGGAACTGAG | |
| G172-DnaE-R | | AGGACAAACATTTGTATAGTTCATCCATGCC | |
| *XbaI*-SC-R | | AGTAACTCTAGAACCAGAACCACAAGAACAAGCTGCAGCAATAGCACCGTTTGCG | |
| *BstB*I-G172C-F | | CCCTTTCGAAAGATCCCAACGA | |
| pUC-HisLV-F | | CGACGTTGTAAAACGACGGCCAGTGCAAGGAGATATAACAATGAAATACCTATTGCCTAC | |
| HisLv-HerLc-R | | GATGAAGACAGATGGCGCCGCCACCGTACGACGTTTTATTTCCAACTTTGT | |
| HcSP-HisHv-F | | TTATTTTTTTCTTAATGGCTGTCGTAACTGGTGTGAATTCCCAGGTTCAGCTGCAGCAAT | |
| HisHv-HerLc-R | | GATGAAGACAGATGGCGCCGCCACCGTACGACGTTTTATTTCCAACTTTGT | |
| pUC-HerL-F | | CGACGTTGTAAAACGACGGCCAGTGCAAGGAGATATAACAATGTTGCCATCACAACTCAT | |
| HerLc-DnaE-R | | TGTAAGTATCTCAGTTCCGAAGGACAAACAACACTCTCCCCTGTTGAAGCT | |
| HerH-F | | GACTGGACCTGGAGGATCCT | |
| HerHFc-pUC-R | | AAACAGCTATGACCATGATTACGCCAAGCTTCATTTACCCGGAGACAGGGAGAG | |
| HerHc-pUC-R | | AAACAGCTATGACCATGATTACGCCAAGCTTCAGGGCTCAACTTTCTTGTCCAC | |
| F2A-HerH-R | | CACCAAGAAGAGGATCCTCCAGGTCCAGTCGGGCCCAGGGTTGGACTC | |
| DnaE-Xho-F-1 | | ACTCCTCGAGTATTGTTTGTCCTTCGGAACTGAGATACTTAC | |

Note: Restriction sites are underlined.

**Supplementary experimental procedures**

**Vector construction**

To assemble the ND-1 polyprotein cassette, the coding sequence of the IntF2A domain was synthesized (Genscript, Piscataway, NJ) and ligated into pUC57 vector between *Xho*I and *Apa*I, to yield pUC-Int(ND/CFN)F2A. The coding sequence of GFP_172_ was amplified from pGEM5z-GFP172 vector using primers *Sal*I-mGFP5 and GFP-*Xho*I-R, to generate the *Sal*I-GFP_172_-*Xho*I fragment. The coding sequence of RFP_Strep_ from pETMD (Han et al., 2012) vector was amplified by two-step PCR with primers (mCherry-2A-F, mCherry-STREP-R and GKZ-R), to generate the *Apa*I-RFP_Strep_-*Sac*I fragment. The *Sal*I-GFP_172_-*Xho*I and *Apa*I-RFP_Strep_-*Sac*I fragments were successively ligated into the pUC-Int(ND/CFN)F2A vector digested with corresponding restriction enzymes, to yield pUC-ND-1. To assemble the three-protein expression cassette (ND-2), *Apa*I-RFP_Strep_-*Sac*I fragment in pUC-ND-1 vector was replaced with the *Apa*I-mKO1_FLAG_-*Sac*I fragment without stop codon which was amplified by two-step PCR from pET-His6-mKO1 using primers mKO1-*Apa*I-F, mKO1-R and FLAG-*Sac*I-R1. The resulting pUC-GFP_172_-Int(ND/CFN)F2A-mKO1_FLAG_ vector was digested with *SacI* enzyme and ligated with the *Sac*I-Int(ND/CFN)F2A-RFP_Strep_-*Sac*I fragment amplified using primers G11c-*Sac*I-DnaE-F and GKZ-R. Orientation of the inserted IntF2A-RFP_Strep_ was screened by colony PCR using primers M13F and G11c-*Sac*I-DnaE-F.

To evaluate different cellular targeting of POIs processed from the IntF2A based polyprotein precursor, a secretory signal peptide was incorporated into the N-terminus of the either POI1 (ND-3) or both POI1 and POI2 (ND-4). In the former case, coding sequence of GFP_His_ with the N-terminal *Arabidopsis thaliana* basic chitinase signal peptide (SP1) was amplified from pBISN1-mgfp5er vector (Haseloff et al., 1997) using primers Sal5er-F and mGFP5-His-*Xho*I-R. An *EcoR*I site was included between SP1 and GFP_His_ sequence to facilitate subsequent cloning steps. The *Sal*I-GFP_His_-*Xho*I fragment was excised from pUC-SP1GFP_His_ vector and ligated into pUC-ND-1 vector after *Sal*I-*Xho*I double enzyme digestion, to yield an intermediate vector, pUC-SP1GFP_His_-Int(ND/CFN)F2A-RFP_Strep_. The pUC-ND-3 vector was generated by replacing the GFP_His_ between *EcoR*I and *Xho*I sites in pUC-SP1GFP_His_-Int(ND/CFN)F2A-RFP_Strep_ vector with GFP_172_ amplified using primers mGFPS2E and GFP-*Xho*I-R. To append ER targeting signal to the N-terminus of POI2, rice α-amylase signal peptide (SP2) {Zhang, 2011 #226}{Zhang, 2011 #226}was first introduced to the N-terminus of RFP_Strep_ by replacing the dsRed-(Kex2)_3_-mGFP5 in pBluescript-RKG vector (Zhang et al., 2011) with *Sph*I-RFP_Strep_-*Sac*I*,* which was amplified from pUC-ND-1 vector using primers mCherry-*Sph*I-F and GKZ-R, to yield pBluescript-SP2-RFP_Strep_. The pUC-ND-4 vector was generated by ligating the SP2-RFP_Strep_ fragment, amplified using primers mCherry-2A-F and GKZ-R, into pUC-ND-3 vector between *Apa*I and *Sac*I sites.

N-terminal cleavage inactive Intein mutant was generated by site-direct mutagenesis using primers (DnaE-*Xho*I-F-2 and DnaE-*Xba*I-R-2) to introduce an Ala mutation at the first Cys residue (C1A mutation) (Xu and Perler, 1996). This N-cleavage inactive intein fragment was used to replace the active DnaE intein between *Xho*I and *Xba*I sites in pUC-ND-1 and pUC-ND-3 vectors, to generate pUC-N(-)-1 and pUC-N(-)-2, respectively.

To create the SC cassettes, the intein sequence was first amplified from the pUC-ND-1 vector using primers G172-DnaE-F and *XbaI*-SC-R and fused to the C-terminus of GFP_172_ by utilizing the translationally silent *BstB*I site near the C-terminal region of GFP_172_. To achieve this, 3’-end region of GFP_172_ sequence was initially amplified using primers *BstB*I-G172C-F and G172-DnaE-R and appended to the 5’-end of the intein fragment by overlapping PCR using primers *BstB*I-G172C-F and *Xba*I-SC-R. The modified intein fragment with partial GFP_172_ sequence was ligated into the pUC-ND-1 vector between *Bst*BI and *Xba*I sites to yield the pUC-SC-1. To create the 2A-1 construct, GFP_172_ with C-terminal GS linker was amplified using primers *Sal*I-mGFP5 and GFP-GS-*Xba*I-R. The resulting *Sal*I-GFP_172_-GS-*Xba*I fragment was ligated into pUC-ND-1 to replace the GFP_172_-Int(ND/CFN) between the *Sal*I and *Xba*I sites.

To create the antibody expression cassette, variable domains of light chain and heavy chain of an single-chain His Tag antibody were amplified from the pAK1Hmut1+2_noM vector (Kaufmann et al., 2002) using primer pairs pUC-HisLV-F/HisLv-HerLc-R and HcSP-HisHv-F/HisHv-HerLc-R, respectively. Constant domains of human IgG1 kappa light chain and human IgG1 heavy chain were respectively amplified from pVITRO1-Trastuzumab IgG1/κ vector (Addgene #61883) using primers pUC-HerL-F/HerLc-DnaE-R and HerH-F/HerHFc-pUC-R. Chimeric light chain and heavy chain of His-Tag antibody were assembled by splicing the variable region and constant region of light chain and heavy chain fragments using overlap extension PCR. The intein-F2A fragment was amplified from the pUC-SC-1 vector using primers DnaE-Xho-F-1 and F2A-HerH-R. Chimeric kappa light chain, IntF2A and heavy chain of His-Tag antibody were assembled in pUC57 vector using NEBuilder HiFi DNA seamless cloning kit (New England Biolab, Ipswich, MA).

For plant expression, the assembled reporter polyprotein cassettes were ligated into the binary vector pE1775 between *Sal*I and *Sac*I sites, in which protein expression in plants was driven by the mannopine/octopine synthase (ocs)_3_/mas promoter (Ni et al., 1995). The *Xho*I and *Spe*I sites were utilized for ligating the IgG polyprotein cassettes into the pE1775 vector. The sequences of all the constructs were verified by DNA sequencing.

**Nucleic acid sequence of the IntF2A domain** (sequence used in SC-1; nucleotides that encode autocleavage-accelerating flanking extein residues Ser and Cys are shown in underline bold letters, cf. Fig. 1)

TGTTTGTCCTTCGGAACTGAGATACTTACAGTTGAATATGGACCACTTCCTATTGGAAAGATTGTGAGTGAAGAGATCAACTGCAGTGTTTATTCCGTGGATCCAGAGGGTAGAGTTTACACTCAAGCAATTGCTCAGTGGCATGATAGGGGAGAACAGGAGGTTCTTGAATATGAGTTGGAAGATGGTTCTGTGATAAGAGCTACATCAGATCACAGGTTTCTTACTACAGATTACCAACTTTTGGCAATCGAAGAGATTTTCGCTAGACAGCTCGATCTTCTCACTTTGGAAAATATTAAGCAAACAGAAGAGGCACTTGATAACCATAGGCTTCCATTTCCTCTTTTGGATGCTGGAACTATTAAGATGGTTAAAGTGATAGGAAGAAGGTCATTGGGTGTTCAAAGAATATTTGATATCGGACTTCCTCAGGATCACAATTTCTTACTCGCAAACGGTGCTATTGCTGCAGCTTGT**TCTTGT**GGTTCTGGTTCTAGAGTTACTGAGCTTTTGTATAGGATGAAGAGGGCAGAAACATACTGCCCAAGACCTTTACTCGCAATCCATCCAACAGAGGCTAGGCACAAGCAAAAAATTGTTGCTCCTGTGAAACAGCTTTTGAACTTTGATCTTCTCAAGCTTGCGGGAGACGTCGAGTCCAACCCTGGGCCC

**References**

Han, Z., Zhang, B., Wang, Y.E., Zuo, Y.Y. and Su, W.W. (2012) Self-assembled amyloid-like oligomeric-cohesin scaffoldin for augmented protein display on the *Saccharomyces cerevisiae* cell surface. *Applied and Environmental Microbiology* **78**, 3249-3255.

Haseloff, J., Siemering, K.R., Prasher, D.C. and Hodge, S. (1997) Removal of a cryptic intron and subcellular localization of green fluorescent protein are required to mark transgenic Arabidopsis plants brightly. *Proceedings of the National Academy of Sciences, USA* **94**, 2122-2127.

Kaufmann, M., Lindner, P., Honegger, A., Blank, K., Tschopp, M., Capitani, G., Plückthun, A. and Grütter, M.G. (2002) Crystal Structure of the Anti-His Tag Antibody 3D5 Single-chain Fragment Complexed to its Antigen. *Journal of Molecular Biology* **318**, 135-147.

Murashige, T. and Skoog, F. (1962) A Revised Medium for Rapid Growth and Bio Assays with Tobacco Tissue Cultures. *Physiologia Plantarum* **15**, 473-497.

Ni, M., Cui, D., Einstein, J., Narasimhulu, S., Vergara, C.E. and Gelvin, S.B. (1995) Strength and tissue specificity of chimeric promoters derived from the octopine and mannopine synthase genes. *The Plant Journal* **7**, 661-676.

Xu, M.Q. and Perler, F.B. (1996) The mechanism of protein splicing and its modulation by mutation. *EMBO Journal* **15**, 5146-5153.

Zhang, B., Rapolu, M., Huang, L. and Su, W.W. (2011) Coordinate expression of multiple proteins in plant cells by exploiting endogenous kex2p-like protease activity. *Plant Biotechnology Journal* **9**, 970-981.
